# Broncho Alveolar Dendritic Cells and Macrophages Are Highly Similar to Their Interstitial Counterparts

**DOI:** 10.1371/journal.pone.0167315

**Published:** 2016-12-19

**Authors:** Pauline Maisonnasse, Elise Bordet, Edwige Bouguyon, Nicolas Bertho

**Affiliations:** Virologie et Immunologie Moléculaires, Institut National de la Recherche Agronomique, Domaine de Vilvert, Jouy-en-Josas, France; Forschungszentrum Borstel Leibniz-Zentrum fur Medizin und Biowissenschaften, GERMANY

## Abstract

In human medicine, bronchoalveolar lavage is the main non-traumatic procedure allowing an insight into the respiratory Dendritic Cells (DC) and Macrophages populations. However, it has never been demonstrated in a relevant model that alveolar DC subpopulations were comparable to their interstitial counterparts. In a precedent work we observed that respiratory pig DC and Macrophages were more similar to the human ones than to the mouse ones. In the present work, thanks to our animal model, we were able to collect the rare bronchoalveolar DC and compare them to their interstitial counterparts. We observed that DC presented very similar gene-expression patterns in the alveolar and interstitial compartments, validating the study of human bronchoalveolar DC as surrogate of their interstitium counterparts.

## Introduction

Dendritic Cells (DC) and Macrophages are an essential part of the respiratory immune system. Their roles in the development and the resolution of respiratory infections are widely studied. They are indeed involved in sensing foreign antigens, controlling inflammation, and initiating the adaptive immune responses.

These myeloid cells can be subdivided in 5 different subsets that we named according to the nomenclature principle proposed by Guilliams *et al*. [1], and used by ourselves in a previous study [2]. This nomenclature is based on the origin and the function of the myeloid cells. Although not officially accepted, it offers the advantage to assign one single name per DC/Macrophages subpopulation for all the species, thus greatly facilitating trans-species comparisons: FLT3-dependent conventional DC (cDC) being Sirpα negative or low and expressing high levels of XCR1 are named cDC1 (BDCA3^pos^ cDC and CD103^pos^ cDC in human and mouse respectively), whereas the Sirpα^high^/CD11b^pos^ cDC are named cDC2 (BDCA1^pos^ and CD11b^pos^ in human and mouse). Monocyte-derived DC (moDC) differentiating upon inflammation are named moDC, whereas monocytes differentiating in Macrophages are called moMacro. Finally, Macrophages originating from embryonic precursors settled in the lung before birth [3, 4] are called Alveolar Macrophages (AM).

Many lung pathologies involve an uncontrolled inflammation. Murine moDC, recruited through the CCR2/CCL2 interaction, are involved in the induction of lung pathological inflammation, for example during an Influenza infection [5]. Human cDC2 accumulation has been observed in lung and airway epithelium during Th2 inflammation-associated asthma [6], while murine cDC2 [7] and moDC [8] accumulated in the lung parenchyma. On the other hand, murine interstitial Macrophages, *bona fide* moMacro, strongly down-modulate the allergic immune response [9].

Those DC/Macrophages populations are thus of great interest in the study of respiratory pathologies, either to boost the immune response or to down-modulate the pathological inflammation.

Porcine and human respiratory systems share several anatomical, histological, physiological, and biochemical elements [10]. Pig is being developed as a model for respiratory pathologies such as Influenza infections [11] or cystic fibrosis [12]. Finally, our team recently characterized porcine DC/Macrophages in lung tissue and tracheal epithelium, and showed their similarities with their human counterparts [2].

In the case of human patients, only the Bronchoalveolar Lavages (BAL) cells are easily available. However, to our knowledge, it has never been formally shown that BAL-collected DC and Macrophages were similar to their parenchymal counterparts.

In order to validate human studies on BAL DC and Macrophages, we though to compare BAL and parenchymal DC/Macrophages in swine.

## Methods

### Animals, in vivo infections, and tissue collection

Tissue samples were obtained from 5- to 7-month-old Large White conventionally bred sows from UEPAO, Tours, France. Animals were euthanized for the normal course of livestock management, in compliance with European directives and with French veterinary authorities’ agreements (agreement number 3717501). They were anesthetized by electro-narcosis, then bled before the collection of the lung and the realization of the BAL. Cells were collected as previously described (Maisonnasse et al. 2015). Briefly, a BAL was performed twice in PBS supplemented with 2mM EDTA (PBS/EDTA) to collect AM. Then, the tissue was dissected, minced and incubated in non-culture treated Petri dishes for two hours at 37°C in complete RPMI, containing 2 mg/ml collagenase D (Roche), 1 mg/ml dispase (InVitrogen) and 0.1 mg/ml Dnase I (Roche). Cells were passed through 40 μm strainers, and red blood cells lysed. Then, cells were washed with PBS/EDTA, counted and step-frozen in FCS plus 10% DMSO (Sigma-Aldrich).

### Flow cytometry analysis and cell sorting

The following Antibodies (Abs) were used: Sirpα/CD172a (74-22-15a) and MHC-II (MSA3) from Monoclonal Antibodies Center Washington State University; CD163 (2A10/11) from AbD-Serotec; isotype-specific secondary reagents coupled to Alexa 488, PE or Alexa 647 from Invitrogen. Cell surface stainings were performed as previously described [2]. Briefly, cells were stained in PBS/EDTA supplemented with 5% horse serum and 5% swine serum for 30 minutes on ice and washed twice with PBS/EDTA between labeling. Samples were acquired on a Fortessa (BD-Bioscience) or sorted on a MoFlo ASTRIOS (Beckman-Coulter). For sorting, preparations were enriched in DC/Macrophages by gradient [13] (Optiprep; Nycomed Pharma) and dead cells were excluded by Dapi staining (Sigma-Aldrich). Acquired data were analyzed using FlowJo software (version X.0.6).

### RNA extraction

Total RNA from sorted cells were extracted using the Arcturus PicoPure RNA Isolation kit according to the manufacturer’s instructions. Contaminating genomic DNA was removed using a Qiagen RNase free DNase set.

### Real-time quantitative PCR (qPCR)

RNA was reverse transcribed using random hexamers and the Multiscribe reverse transcriptase (Life Technologies). qPCR were performed as previously described [2]. Briefly, reactions were carried out with 300 nM primers in a final reaction volume of 25 μl of 1 X SYBR Green PCR Master Mix (Applied Biosystems). PCR cycling conditions were 95°C for 10 min, linked to 40 cycles of 95°C for 15 s and 60°C for 1 min. Real-time qPCR data were collected by the Mastercycler^®^ e0p realplex-Eppendorf system and 2−ΔCt calculations for the relative expression of the different genes (arbitrary units) were performed with the Realplex software using ovRPS24 (ovine Ribosomal Protein S24) as reference gene. This gene has been carefully chosen after a transcriptomic screen (data not shown). RPS24 expression being more stable than HPRT, RPL19 and GAPDH expressions, when tested on the different DC/Macrophages populations in different animals, and upon influenza infection.

The primers used were: ovRPS24 (F: AAGGAACGCAAGAACAGAATGAA, R: TTTGCCAGCACCAACGTTG); FLT3 (F: TGTTCACGCTGAATATAAGAAGGAA, R: GGAGCAGGAAGCCTGACTTG); XCR1 (F: CGATGCCGTCTTCCACAAG, R: GGAACCACTGGCGTTCTGA); FCεR1α (F: AATTTACAGACCCACAGCCTAGCT, R: TGCTATCGCAGATGTTTCTTGAG); CD103 (F: GATGCGGAACATCTATGAGAAGTG, R: CTGGATGACGCTCCCGTATT); cKit (F: TGGGCTCGAGAAGTCAAGTATTT, R: ATGCCCGGAGAGCATTTTT); CCR2 (F: ACACGCTTTCCCGGTTCA, R: CCCTTGATATTCATTGTAAGCAGAGA); CX3CR1 (F: CGTGGCCCTGGGAACTG, R: CGAGGCCAAAGGCAAAAA); CSF1R (F: TGAACGACTCCAACTACATTGTCA, R: TGTAGACGCAGTCGAAGATGCT); MerTK (F: CCGAACTCTGTAATCGCTTCTTG, R: TGCACTTCCGCCGTGACTA); MAFB (F: TGCGTTCTTTAGACCAATATGTTATGT, R: CACCAATAACTCGCCCGCTAT).

### Statistical analysis

All data were analyzed using the GraphPadPrism v5.0 statistical software package (GraphPad Software, La Jolla, CA). Statistical tests applied to each data set are indicated in the relevant figure legend.

## Results and Discussion

**Fig 1:**
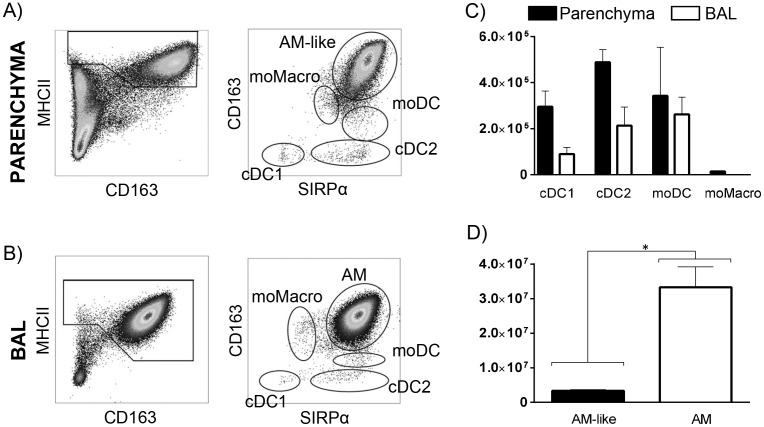
Bronchoalveolar lavage (BAL) and lung interstitial (parenchyma) cells were obtained from the diaphragmatic lobe (A and B) or the right cardiac lobe (C and D) and stained for multi-color flow cytometry. (A and B) gating strategy used for the count and cell sorting of each subpopulation as previously described [2]. (C and D) Absolute number of each subset among total MHCII^high^ cells was assessed in parenchyma and BAL (n = 4). cDC = conventional Dendritic Cells, moDC = monocyte-derived DC, moMacro = monocyte-derived Macrophages, AM = alveolar Macrophages. Statistic signs *: P<0.01, **: P<0.001 with a Mann-Whitney test.

We segregated DC and Macrophages using a staining and gating strategy as described in Fig 1A and 1B, according to our previous work [2], in which we precisely defined phenotypically and functionally these different parenchymal subpopulations as well as the alveolar macrophages. In short, we stained parenchymal lung cells or BAL cells for MHC-II, CD163 and Sirpα. Among parenchymal MHC-II^high^ cells, Sirpα^high^/CD163^high^ cells were defined as AM-like cells, Sirpα^interm^/CD163^interm^ cells were moMacro, Sirpα^high^/CD163^low^ cells were moDC, Sirpα^high^/CD163^neg^ cells were cDC2 and Sirpα^neg^/CD163^neg^ cells were cDC1. In the BAL, because of their strong prevalence, we previously described the only Sirpα^high^/CD163^high^ AM population, although rare cells could be observed presenting phenotypes related to the moMacro, moDC, cDC1 and cDC2 parenchymal cells (Fig 1B). Here, we first assessed the number of each DC/Macrophages populations in the parenchyma and BAL of 4 pigs. In order to compare the absolute number of cells in the alveoli and tissue, we worked on the right cardiac lobe which is small enough to collect the whole tissue.

It appeared that cDC2 and cDC1 were 2 and 3 times less numerous in the alveoli than in the tissue (Fig 1C), with 213.10^3^ cDC2 and 89.10^3^ cDC1 in the BAL to be compared with 489.10^3^ cDC2 and 295.10^3^ cDC1 in the parenchyma. In the BAL, Sirpα^interm^/CD163^interm^ cells, first identified as moMacro, presented a gene expression pattern identical to AM (data not shown), so they were pooled with the Sirpα^high^/CD163^high^ AM population. Thus, moMacro could not be identified in BAL, probably due to their absence or their very low proportion compared with AM. Indeed, in the parenchyma, not more than 14.10^3^ moMacro cells/lobe were counted. The moDC were equally present in BAL and parenchyma (respectively 262.10^3^ and 343.10^3^ per lobe). However, the main MHCII^high^ populations in the lung were AM (33.10^6^ cells per lobe) and AM-like cells (3.10^6^ cells per lobe), the latter being 10 times less represented than AM (Fig 1D). AM-like cells still represented the main population in the interstitium and were at least 5 times more numerous than the other 4 populations we observed there.

**Fig 2:**
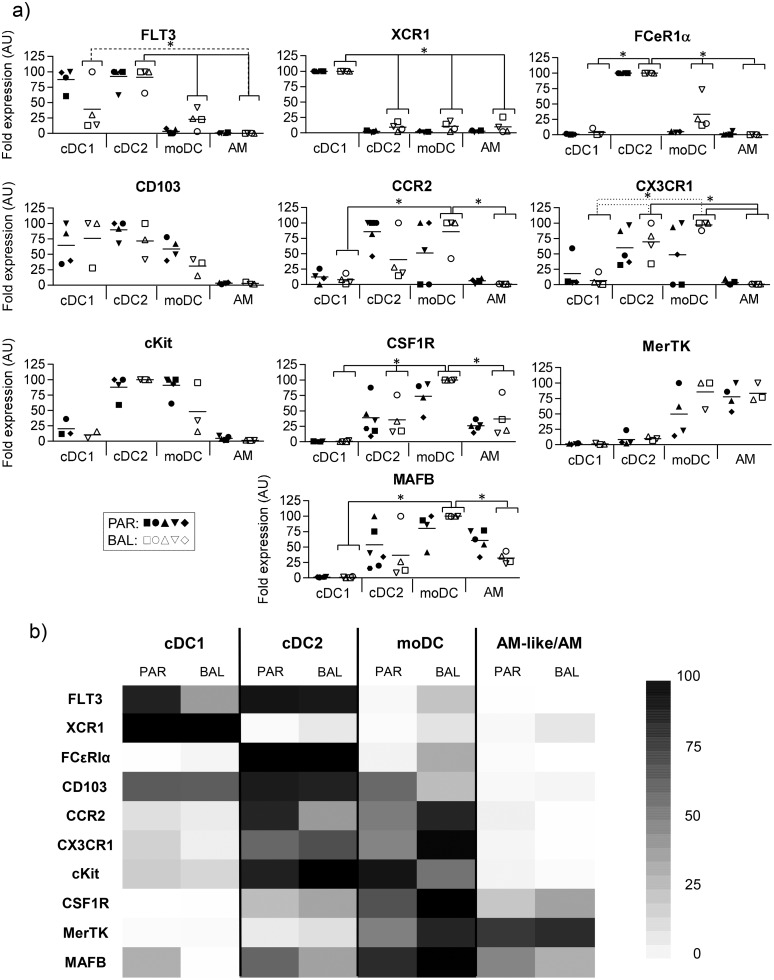
(A) The different subsets from BAL were sorted by flow cytometry. mRNA expression levels of 10 genes were assessed by RT-qPCR. For each gene, data were normalized to the reference gene RPS24 (Ribosomal Protein S24) expression and presented as relative expression (arbitrary units (AU)): for each animal, the population with the highest expression was considered as 100 and the other populations were normalized to it. Each symbol represents one animal. For the sake of an easier comparison between parenchymal (closed symbols) and BAL (open symbols) populations, parenchymal cells data (closed symbols), previously published in [2] were integrated in this figure. B) Heat map depicting the results from 2A. cDC = conventional Dendritic Cells, moDC = monocyte-derived DC, moMacro = monocyte-derived Macrophages, AM = alveolar Macrophages. Statistic signs *: P<0.01, **: P<0.001 with Student t-test.

The BAL cells were then sorted by flow cytometry and the transcriptomic expressions of 10 genes previously identified as differentially expressed in these populations by us [2, 14] and others [4, 15, 16] were measured by RT-qPCR (Fig 2A). In order to easily compare those results with those we previously obtained, we depicted previously published data from parenchymal cells [2] as closed symbols in Fig 2A and as the “PAR” columns in the heat map (Fig 2B) we designed to summarize the RT-qPCR data. The two cDC populations were highly similar between BAL and tissue, expressing both high levels of Flt3, whereas cDC1 specifically expressed XCR1 and cDC2 specifically expressed FCεR1α. BAL moDC clearly expressed the monocytic genes CSF1R, MerTK and MAFB, proving their belonging to the moDC subset. The main differences, although not significant, with their interstitial counterpart resided in their higher expressions of CCR2 and CX3CR1. Finally, and as we had previously shown, AM-like cells were highly similar to AM. They had a strong expression of the Macrophage-associated gene MerTK, and low expressions of genes which are specific of hematopoietic cells such as cKit, CCR2 or CX3CR1, in agreement with an origin independent from the bone marrow. Interestingly, only one gene expression appeared different, although not significant, between AM and AM-like cells, AM-like cells expressing more MAFB than AM.

To conclude, we showed here that in the swine model, BAL DC/Macrophages appear similar to their interstitial counterparts, with a probable common origin and similar differentiation patterns. However, their establishment in different lung compartment may significantly influence their functions, which still need to be investigated.
