# Correction: Broncho Alveolar Dendritic Cells and Macrophages Are Highly Similar to Their Interstitial Counterparts

**DOI:** 10.1371/journal.pone.0171852

**Published:** 2017-02-06

**Authors:** The PLOS ONE Staff

There is an error in the Funding statement. The name of the funder Fondation du soufflé is incorrect. The correct name is: Fondation du Souffle. The publisher apologizes for the error.
